# Pareto optimization with small data by learning across common objective spaces

**DOI:** 10.1038/s41598-023-33414-6

**Published:** 2023-05-15

**Authors:** Chin Sheng Tan, Abhishek Gupta, Yew-Soon Ong, Mahardhika Pratama, Puay Siew Tan, Siew Kei Lam

**Affiliations:** 1 Agency for Science, Technology and Research (A*STAR), Singapore, Singapore; 2 Singapore Institute of Manufacturing Technology (SIMTech), Singapore, Singapore; 3 School of Computer Science and Engineering, Nanyang Technological University, Singapore, Singapore; 4 STEM, University of South Australia, Adelaide, Australia

**Keywords:** Engineering, Mathematics and computing

## Abstract

In multi-objective optimization, it becomes prohibitively difficult to cover the Pareto front (PF) as the number of points scales exponentially with the dimensionality of the objective space. The challenge is exacerbated in expensive optimization domains where evaluation data is at a premium. To overcome insufficient representations of PFs, *Pareto estimation* (PE) invokes inverse machine learning to map preferred but unexplored regions along the front to the Pareto set in decision space. However, the accuracy of the inverse model depends on the training data, which is inherently scarce/small given high-dimensional/expensive objectives. To alleviate this small data challenge, this paper marks a first study on multi-source inverse transfer learning for PE. A method to maximally utilize experiential *source* tasks to augment PE in the *target* optimization task is proposed. Information transfers between heterogeneous source-target pairs is uniquely enabled in the inverse setting through the unification provided by common objective spaces. Our approach is tested experimentally on benchmark functions as well as on high-fidelity, multidisciplinary simulation data of composite materials manufacturing processes, revealing significant gains to the predictive accuracy and PF approximation capacity of Pareto set learning. With such accurate inverse models made feasible, a future of on-demand human-machine interaction facilitating multi-objective decisions is envisioned.

## Introduction

Multi-objective optimization problems (MOPs) involve a search for decision variable values that, without loss of generality, minimize a *set* of objective functions. Such problems find wide applicability in a range of real-world settings, including in engineering^1,2^, economics^3,4^, logistics systems planning^5,6^, manufacturing operations optimization^7,8^, to name just a few. In a non-trivial setting, the objective functions conflict with one another, such that no single solution exists that can simultaneously minimize all of them. The focus is then to search for a set of optimal trade-off solutions, those for which some objective(s) can be improved but only by worsening some other objective. The set of all such solutions constitutes the Pareto set (PS) in decision space, whose image in objective space forms what is referred to as the Pareto front (PF)^9^. Uncovering the PF shall provide a decision maker (DM) with a comprehensive view of all possible trade-offs, allowing her to select a solution *a posteriori* based on her preferences. The goal of an MOP solver is then to efficiently arrive at a good approximation (in terms of both convergence and coverage) of the entire PF.

In the literature, MOPs have been tackled using exact^10–12^ and approximate sampling-based methods^13–15^ that typically produce discrete representations of possibly continuous PFs. One common procedure is to decompose an MOP into a set of single-objective optimization sub-problems, which are then jointly solved to produce a corresponding set of near-optimal trade-off solutions^16^. Alternative approaches that simultaneously evolve populations of solutions towards diverse regions of the PF without the need for explicit problem decomposition are also popular in practice^17,18^. In most cases however, the total number of points (solutions) needed to achieve good coverage of the PF scales exponentially with the number of objective functions^19^. This renders many existing approaches intractable as the dimensionality of the objective space increases. The challenge is further exacerbated in expensive optimization domains (e.g., those requiring time-consuming computer simulations or complex real-world procedures for function evaluation), where evaluation data is at a premium. As a result, points preferred by the DM may not be sufficiently represented in the obtained sparse PF approximation.

A promising approach to enhance the density of PF approximation is to train an inverse machine learning model to map points from the front to the decision space^20^, with training carried out on data acquired from a run of any MOP solver. Assuming a “perfect” inverse model in hand, *Pareto estimation* (PE) can then be performed to generate new solutions in the PS corresponding to any arbitrary unexplored sub-region of the PF^21^. This possibility hints to a future of seamless human-machine interaction in multi-objective decision-making, where a DM is able to arrive at desired solutions on-demand by simply querying the model with preferred trade-offs in objective space. However, even in the context of PE, the curse of dimensionality rears its ugly head as the accuracy of the inverse model is itself dependent on the quality and quantity of available training data, which is inherently scarce/small in high-dimensional/expensive optimization domains.

To alleviate this small data challenge, this paper marks a first study on *multi-source inverse transfer learning* for PE. Optimization problems seldom exist in isolation, especially in industrial setups where similar problems routinely recur^22^. Therefore, there often exist experiential *source* tasks whose data could potentially be utilized to augment inverse modeling in the *target* MOP. The inverse machine learning setting allows one to uniquely leverage data from heterogeneous source MOPs as well, whose decision space may differ from that of the target (e.g., decision variables could be added or removed in the target relative to the source^23^). This possibility arises from observing that objective functions of interest frequently coincide in MOPs belonging to a particular application area, even if the decision variables change across tasks. *The common objective space (which serves as the input to the inverse model) thus provides the necessary unification for information transfers to occur between otherwise heterogeneous source-target pairs*. An exemplar of this is shown in our engineering case-study, where although different composite part manufacturing processes possess differing decision variables, the objective functions pertaining to part quality, throughput, and peripheral equipment costs remain the same^24^.

The proposed method builds on probabilistic Gaussian process (GP)^25^ inverse models. A strong motivation behind this choice is the uncertainty-awareness of GPs, deemed invaluable for rationalizable human-machine interactions^26^. Our method adapts the transfer GP (TGP) model^27^ to the inverse machine learning setting, giving a separate inverse TGP (*inv*TGP) for each source-target pair. Assuming $\gamma$ source MOPs, the resulting $\gamma$ *inv*TGPs are then fused by means of a scalable generalized product-of-experts model^28,29^. A salient feature of the product-of-experts is that it constructs solutions in decision space by composing decision variable values according to each *inv*TGP’s predictive uncertainty. Low predicted variances (indicating confident predictions) are more strongly weighted, leading to a confident fused prediction. This result shall be explained in some detail in section “Product-of-*inv*TGPs for multi-source transfer”.

In summary, the main contributions of this paper are as follows.

- A novel multi-source inverse transfer learning method (a generalized product-of-*inv*TGPs) is put forward for PE. The method harnesses scarce/small datasets generated in high-dimensional/expensive MOPs where an optimization algorithm is only able to produce a sparse representation of the PF. A future of on-demand human-machine interaction in multi-objective decision-making is envisioned by means of accurate inverse modeling.
- The approach uniquely exploits our observation that common objective spaces frequently occur in MOPs belonging to a given application area. In the inverse machine learning setting, this provides the necessary unification for information transfers to take place even between heterogeneous source-target task pairs.
- The performance of the generalized product-of-*inv*TGPs is verified on multi-objective benchmark functions. The results show that the accuracy of PF approximation can be twice as high ($\sim$50% lower error) as standard no-transfer PE under data scarcity. Similarly, when applied to expensive simulation data from the design optimization of composites manufacturing processes, an improvement of up to $\sim$17% in predictive accuracy of Pareto set learning is achieved.

The remainder of the paper is organized as follows. In section “Related work”, we briefly review the literature on works associated with the concept of PE. Section “Preliminaries” presents technical background on multi-objective optimization and inverse machine learning for mapping the PF in objective to the PS in decision space. Section “Harnessing small datasets in pareto set learning” introduces the methodology and rationale behind multi-source inverse transfer learning as put forward in this paper. Section “Empirical analysis” carries out a rigorous experimental study of the method on benchmark MOPs with 4-D to 7-D objective spaces and on a composites manufacturing use-case. Finally, Section “Conclusion” closes the paper with a recap of the main ideas and future research outlooks.

## Related work

In this section, we briefly review existing work associated with the topic of Pareto estimation (PE). The literature is broadly categorized into two research strands, referred to herein as (a) *post-hoc PE* and (b) *online PE*, with the former being the main focus of this paper.

Given a target MOP, and given solution evaluation data generated in the course of *a posteriori* multi-objective optimization, post-hoc PE serves to aid decision-making by enhancing the density of the PF approximation. This is achieved via inverse models that can map points from the objective to the decision space. The goal is for a DM to be able to generate new near-optimal solutions on-demand, simply by querying the inverse model at unexplored regions of the PF. An early work in this regard was carried out by Giagkiozis and Fleming^21^, where they employed an inverse radial basis function network (labelled hereafter as *inv*RBFNN) for post-hoc PE. While their method was agnostic to the choice and behaviour of the underlying MOP solver, subsequent attempts to improve the accuracy of the *inv*RBFNN have sought to refine the distribution/placement of training samples generated during the optimization run. One generally applicable idea, not restricted to *inv*RBFNNs, is to bias the optimizer to generate more data in regions of greater geometrical change in the PS^30^, under the intuitive assumption that the topology of a function can be interpolated better if its high variation regions are well sampled.

Other works in post-hoc PE have considered challenges stemming from complexities of the PF. For example, Kudikala et al.^31^ proposed a method for multi-modal MOPs, where a one-to-many mapping could arise from objective to decision space due to the presence of multiple solutions that result in identical objective function values along the PF. Gupta et al.^20^ investigated PE for many-objective optimization problems (MaOPs: those with four or more objective functions). The authors revealed a blessing of dimensionality of many-objective search, showing that training data generated from an MaOP could result in better PE accuracy compared to the data generated from its dimensionally reduced counterpart. In a more recent work, Yu et al.^32^ proposed an algorithm for detecting knee regions (that are naturally preferred by DMs) along high-dimensional/complex PFs, facilitating the discovery of corresponding points in the PS by an *inv*RBFNN.

In addition to aiding post-hoc decision-making, online PE influences the workings of the optimization algorithm itself. Some of the latest examples of this include neural Pareto set learning in multi-objective combinatorial optimization^33^, or in multi-objective Bayesian optimization of computationally expensive problems^34^. Here, the inverse models are repeatedly updated based on data being generated during an optimization run, and subsequently inform the sampling of promising solution candidates in the next iterations. To this end, Cheng et al.^35^ utilized multiple inverse GPs (labelled hereafter as *inv*GPs). Each *inv*GP was tasked to predict a single decision variable value. The training data was first partitioned into subspaces in objective space based on uniformly distributed reference vectors. Within a subspace, they then applied a random grouping technique to determine which inverse models were to be built, training an *inv*GP for each.

To address the issue of irregular (non-uniform or disconnected) PFs, various objective space partitioning techniques have also been proposed in the literature. Adaptive reference vector generation in the context of online PE was explored by Cheng et al.^36^, adjusting or removing reference vectors based on the number of solutions associated with each partition. *K*-means clustering was applied by Farias and Araújo^37^ to partition the data before training multiple inverse models. Alternatively, the random grouping mechanism by Cheng et al.^35^ has been the subject of further study and refinement. For instance, a feature importance method with random forests^38^ was applied to determine better assignments of decision variables to objective functions. Likewise, a nonrandom grouping strategy^39^ was put forth to enhance the reliability of the inverse model.

Despite the growing interest in both post-hoc and online PE, we find that research in these areas is still in a nascent state relative to the myriad of multi-objective optimization algorithms with forward models^40^. With that in mind, this paper marks a first step in introducing multi-source inverse transfer learning to post-hoc PE, with a focus on applications in small data regimes. Throughout the remainder of this work, no restriction is placed on the workings of the underlying MOP solver. The paper thus opens new avenues for seamless human-machine interactions at the multi-objective decision-making stage, encompassing problems with high-dimensional/expensive objectives. Use-cases exist in dynamic MOPs as well, where trained inverse models can be used to generate solutions that warm-start the search in changing optimization environments, akin to the work by Zhang et al.^41^. In the future, we foresee such transfer learning-enabled PE to be coupled with MOP solvers even in the online mode, possibly giving rise to new kinds of multi-objective *transfer optimization* algorithms^42,43^.

## Preliminaries

In this section, we present the basics of multi-objective optimization, definitions of its key concepts, and an overview of the steps involved in post-hoc PE.

### Multi-objective optimization

A multi-objective minimization problem can be stated as follows,

1 $$\begin{aligned} &\min _{{\textbf {x}}} \;\;\; {{\textbf {f}}}({{\textbf {x}}}) = [f_1({{\textbf {x}}}), f_2({{\textbf {x}}}),\ldots, f_m({{\textbf {x}}})]\\&\quad s.t. \;\;\; {{\textbf {x}}}\in {\mathcal {X}}\subset {\mathbb {R}}^d, \end{aligned}$$

where *m* is the total number of objectives to be minimized, $f_i$ being the $i^{th}$ objective function, and ${\mathcal {X}}$ being the feasible region of a *d*-dimensional decision space. ${{\textbf {f}}}({{\textbf {x}}})$ is thus a *forward map* from points in decision space to the objective space. Note that a maximization problem could simply be written as minimizing the negative of ${{\textbf {f}}}({{\textbf {x}}})$.

Assuming conflicting objectives in Eq. (1) (such that no single solution exists that simultaneously optimizes all the objectives), the goal is to arrive at a *set* of so-called *Pareto optimal* solutions, with each solution embodying a different trade-off among the objectives. Below, we provide definitions of key terms associated with the notion of Pareto optimality in MOPs^44^.

#### Definition 1

(Pareto Dominance) A solution ${\textbf {x}}_a$ is said to Pareto dominate solution ${\textbf {x}}_b$ if $\forall i \in \{1,2,..,m\}$: $f_i({\textbf {x}}_a) \le f_i({\textbf {x}}_b)$ and $\exists j \in \{1,2,..,m\}$ such that $f_j(x_a) < f_j(x_b)$.

#### Definition 2

(Pareto Optimality) A solution ${\textbf {x}}^*$ is called Pareto optimal if there exists no solution ${\textbf {x}}$ that Pareto dominates ${\textbf {x}}^*$.

#### Definition 3

(Pareto Set) The set of all Pareto optimal solutions constitutes the Pareto set (PS) in decision space.

#### Definition 4

(Pareto Front) The image of the Pareto set in the objective function space is called the Pareto font (PF).

#### Definition 5

(Ideal Point) The ideal point is the vector in objective space whose components are the solution of each single-objective problem $\min _{{{\textbf {x}}}\in {\mathcal {X}}} f_i({{\textbf {x}}})$, $i = 1, 2, \dots , m$.

#### Definition 6

(Nadir Point) The nadir point is the vector in objective space whose components are the solution of each single-objective problem $\max _{{{\textbf {x}}}\in {\mathcal {X}}_P} f_i({{\textbf {x}}})$, $i = 1, 2, \dots , m$, where ${\mathcal {X}}_P$ denotes the PS.

These concepts lie at the heart of PE where we wish to obtain an accurate inverse map from the PF in objective space to the PS in decision space. In this regard, the ideal and nadir points provide the lower and upper bound vectors that constrain the set of possible points in the objective space.

### Pareto set learning for pareto estimation

In post-hoc PE, no strong assumption is made about the algorithm used to solve Eq. (1). Let the PF approximation data generated by the end of a run of any MOP solver be $Y \in {\mathbb {R}}^{n \times m}$, and the corresponding non-dominated solutions in decision space be $X \in {\mathbb {R}}^{n \times d}$, where *n* is the number of points generated along the PF. For optimization in domains with expensive objective functions, *n* would typically be small—e.g., in the order of hundreds or fewer points^15^—offering insufficient coverage of the PF. Likewise, in problems with high-dimensional objective spaces, generating enough Pareto optimal solutions to cover the entire PF becomes computationally intractable. In such cases, PE can serve to enhance the density of the PF approximation, or satisfy a DM’s postponed preferences by generating optimized solutions on-demand in the PS^21^.

However, for a DM to precisely articulate her preferences along an approximated PF, its topology should be known. This is inherently difficult given our initial assumption of data scarcity. Moreover, MOPs with complex, irregular PFs (such as those with discontinuities) add to the difficulty. Hence, the first step towards post-hoc PE is to transform points along the approximated PF *Y* into a projected set $W \in {\mathbb {R}}^{n \times m}$ that can be queried independently of the PF’s topology. The transformation maps each point in *Y* to a point in *W*, which we denote by the function,

2 $$\begin{aligned} \Pi ^{-1}: Y \rightarrow W. \end{aligned}$$

**Figure 1:**
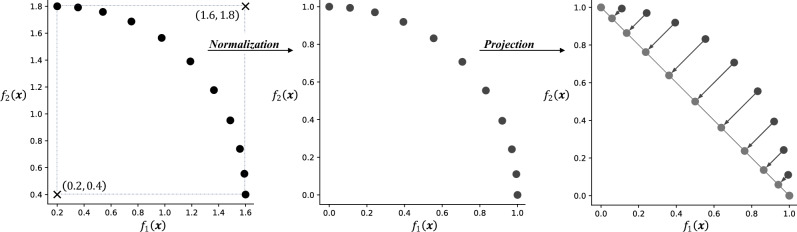
Illustration of the $\Pi ^{-1}$ mapping procedure from the approximated PF *Y* (black dots) to the projected set *W* (grey dots) along the unit hyperplane.

Figure 1 illustrates one such procedure for *m* = 2. The data in *Y* is first normalized to the range [0, 1] based on the ideal and nadir points estimated from *Y*. The normalized points then undergo orthogonal projection onto the unit hyperplane ${\mathcal {W}}$ to produce the dataset *W*. The hyperplane is defined by the (*m*-1)-simplex $\{{{\textbf {e}}}_1,\ldots, {{\textbf {e}}}_{m}\}$, where ${{\textbf {e}}}_i$ is a vector of zeros with a one in the $i^{th}$ position. In the case of Fig. 1, the hyperplane reduces to a line passing through (0, 1) and (1, 0), along which the DM can more easily articulate her preferences for $f_1$ or $f_2$ or a weighted combination of them, without having to deeply take into consideration the topology of the PF.

Given the projected set of points, PS learning entails the training of an inverse machine learning model $\varvec{\psi }^{-1}_{\varvec{\theta }}$, parameterized by $\varvec{\theta }$, on the derived dataset $D = \{W, X\}$. Points in *W* serve as the inputs to the inverse model and those in *X* serve as its outputs for supervised learning; i.e., $\varvec{\psi }^{-1}_{\varvec{\theta }}: {\mathcal {W}} \rightarrow {\mathcal {X}}$. With an accurate inverse model in hand, a DM can in principle query the model with an arbitrary set of points $W_q \subset {\mathcal {W}}$ in unexplored sub-regions of the projected PF, producing desired solutions in the PS as,

3 $$\begin{aligned} \varvec{\psi }^{-1}_{\varvec{\theta }}(W_q) = X_q. \end{aligned}$$

The solutions in $X_q$ can then be evaluated with the forward map to validate the quality of outputs produced by the inverse model. For example, the model’s PF approximation capacity can be quantified by the improvement in spread and convergence to the PF of $Y_q = {{\textbf {f}}}(X_q)$ relative to the points used for training. (For synthetic problems where the theoretical PF is known, this can be achieved by means of various *generational distance* metrics^45^.) Assuming a smooth one-to-one mapping between the PS and the (*m*-1)-dimensional unit hyperplane in objective space^35^, the accuracy of the inverse model to a specific DM query could also be quantified by the Euclidean distance of its prediction to the true Pareto optimal solution.

**Figure 2:**
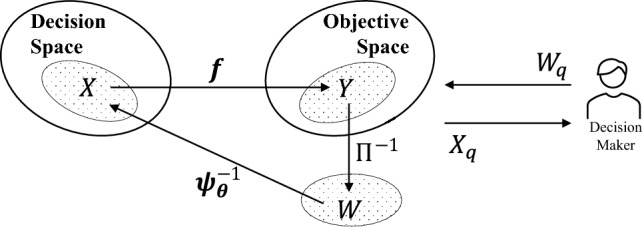
The PE workflow. The inverse machine learning model can be queried with arbitrary preference vectors $W_q$ by the DM to get predicted solutions $X_q$ in the PS.

A schematic of the workflow of post-hoc PE, together with a DM in the loop, is depicted in Fig. 2. It is worth emphasising that if the Karush-Kuhn-Tucker conditions hold in a given problem, then both the PF and PS are (*m*-1)-dimensional piecewise continuous manifolds for *m*-objective optimization problems under certain mild conditions. This has led to the common assumption, albeit without guarantee, that the mapping from the PF to PS is indeed a one-to-one injective function^35,46^. Injectivity justifies the inverse modeling approach in theory. It has however been postulated that even in practice, non-injectivity does not delimit post-hoc PE and could in fact be rather helpful for inverse modeling^21^.

## Harnessing small datasets in pareto set learning

An accurate inverse model can offer significant benefits to a DM in controlled generation of desired PS solutions. However, the accuracy of $\varvec{\psi }^{-1}_{\varvec{\theta }}$ depends on the quality and quantity of available training data, which is inherently scarce/small in high-dimensional/expensive objective spaces. Hence, in this section, we propose to overcome the challenge of limited data regimes via a novel *inverse transfer learning* method.

Consider $\gamma$ source datasets $\{D_{{\mathcal {S}}_1},\ldots,D_{{\mathcal {S}}_\gamma }\}$ with $D_{{\mathcal {S}}_k}=\{W_{{\mathcal {S}}_k},$ $X_{{\mathcal {S}}_k}\}$, $\forall k = 1,\ldots, \gamma$, alongside target data $D_{\mathcal {T}}=\{W_{\mathcal {T}}, X_{\mathcal {T}}\}$ derived from the optimization task at hand. It is assumed that these datasets originate from varied but related MOPs within a given application area, such that the unit hyperplanes containing $W_{{\mathcal {S}}_k} \in {\mathbb {R}}^{n_{{\mathcal {S}}_k}\times m}$ and $W_{\mathcal {T}} \in {\mathbb {R}}^{n_{\mathcal {T}} \times m}$ may lie in a common objective space; i.e., ${\mathcal {W}}_{{\mathcal {S}}_k} = \mathcal {W_{T}}$, $\forall k$. (A real-world exemplar of this is presented in section “Empirical analysis”.) Given high-dimensional/expensive objectives, the target data is inevitably sparse or small, whereas a sizeable *cumulative* volume of source data is deemed available from past problems solved (i.e., $n_{\mathcal {T}}<< \sum _{k=1}^\gamma n_{{\mathcal {S}}_k}$ even if each $n_{{\mathcal {S}}_k}$ may be small). This motivates maximal utilization of information from the experiential sources to augment target PE.

Crucially, PS learning through a common objective space allows for information transfers in scenarios where the decision spaces ${\mathcal {X}}_{{\mathcal {S}}_k}\subset {\mathbb {R}}^{d_{{\mathcal {S}}_k}}$ and ${\mathcal {X}}_{\mathcal {T}}\subset {\mathbb {R}}^{d_{{\mathcal {T}}}}$ of a source and target task may differ. In particular, the dimensionality of the space could change (i.e., $d_{{\mathcal {S}}_k} \ne d_{\mathcal {T}}$) with some decision variables/dimensions being added (or removed) in the target MOP relative to the source^23^. The common objectives (which form the inputs to the inverse model) provide the necessary unification for transfer learning to occur even between such heterogeneous source-target pairs. For practicality, our proposed inverse learner models each decision variable independently; a useful implication of this is given in Section “Product-of-*inv*TGPs for multi-source transfer”. Inverse transfer learning is activated *only* between those source and target decision variables that bear the same physical meaning. We leverage this assumption to condense the exposition in subsequent subsections to only a single (the $j^{th}$) target variable $x_{{\mathcal {T}},j}$. An overlapping source decision variable bearing the same physical meaning is denoted as $x_{{\mathcal {S}}_{k},j}$.

### Inverse TGPs for single-source transfer

First consider standard (no-transfer) PS learning with stochastic, nonparametric GPs. Let the target data be $D_{{\mathcal {T}},j} =\{W_{\mathcal {T}}, X_{{\mathcal {T}},j}\}$ where $X_{{\mathcal {T}},j}$ represents the $j^{th}$ column of $X_{\mathcal {T}}$. In this case, an *inv*GP model, from ${{\textbf {w}}} \in {\mathcal {W}}$ to $x_{{\mathcal {T}},j} \in {\mathbb {R}}$, describes a distribution over functions as $\psi ^{-1}({{\textbf {w}}}) \sim \mathcal{G}\mathcal{P}(\mu ({{\textbf {w}}}), k({{\textbf {w}}},{{\textbf {w}}}'))$, where $\mu ({{\textbf {w}}})$ is the mean (typically set to a constant, zero) and $k(\cdot ,\cdot )$ is some valid covariance function. The inverse map is thus a stochastic process wherein any finite subset of random variables follows a joint multivariate Gaussian distribution. Given the observations in $D_{{\mathcal {T}},j}$, the posterior predictive distribution at any query point ${{\textbf {w}}}_q$ can then be analytically obtained^25^.

In the transfer learning setting with a single source dataset $D_{{\mathcal {S}},j}=\{ W_{\mathcal {S}}, X_{{\mathcal {S}},j}\}$, an *inv*TGP model can account for the similarity between the source and target tasks by extending the covariance function $k(\cdot , \cdot )$ as,

4 $$\begin{aligned} {\tilde{k}}_j({{\textbf {w}}},{{\textbf {w}}}')= {\left\{ \begin{array}{ll} \lambda _j k({{\textbf {w}}}, {{\textbf {w}}}'), &{} \text {if } {{\textbf {w}}} \in W_{{\mathcal {S}}}\, \& \,{{\textbf {w}}}' \in W_{{\mathcal {T}}} \\ &{} \text {or } {{\textbf {w}}} \in W_{{\mathcal {T}}}\, \& \,{{\textbf {w}}}' \in W_{{\mathcal {S}}} \\ k({{\textbf {w}}}, {{\textbf {w}}}'), &{} otherwise \end{array}\right. }, \end{aligned}$$

where ${\tilde{k}}_j(\cdot , \cdot )$ is referred to as the transfer kernel. $\lambda _j$ is a measure of source-target correlation, with $|\lambda _j| \le 1$ being a sufficient condition for the transfer kernel to be valid. As such, if $|\lambda _j|$ is learnt to be close to 1, it indicates high relevance of the source to the target task, whereas $\lambda _j$ close to zero signifies that the source may be unrelated to the target. In the geostatistics literature, this model corresponds to the *intrinsic coregionalization model*, a specific case of *co-kriging* that uses only a single (scalar) $\lambda$ to capture the inter-task similarity^47^. In contrast, the *linear model of coregionalization* from geostatistics may offer greater flexibility by using multiple kernels, but at the added cost of complicating model training and inference^48^. We therefore limit our implementation here to a scalar $\lambda$, achieving encouraging performance as shown in the experiments.

For posterior inference, the closed-form predicted mean and variance of the *inv*TGP at a query point ${{\textbf {w}}}_q$ is given by,

5a $$\begin{aligned} \mu _j({{\textbf {w}}}_q)&= \tilde{{{\textbf {k}}}}_{{\textbf {{w}}}_q}({\tilde{K}} + \Lambda )^{-1} \begin{bmatrix}X_{{\mathcal {S}},j}\\ X_{{\mathcal {T}},j}\end{bmatrix}, \end{aligned}$$

5b $$\begin{aligned} \sigma _j^2({{\textbf {w}}}_q)&= {\tilde{k}}({{\textbf {w}}}_q, {{\textbf {w}}}_q) - \tilde{{{\textbf {k}}}}_{{{\textbf {w}}}_q}({\tilde{K}} + \Lambda )^{-1} \tilde{{{\textbf {k}}}}_{{{{\textbf {w}}}}_q}^\intercal , \end{aligned}$$

where $\tilde{{{\textbf {k}}}}_{{{\textbf {w}}}_q}$ is the kernel vector between ${{\textbf {w}}}_q$ and $W = \{W_{{\mathcal {S}}}, W_{{\mathcal {T}}}\}$ computed using the transfer kernel in Eq. (4), $$\Lambda = \begin{bmatrix} \sigma ^2_{\mathcal {S}} I_{n_{\mathcal {S}}}, &{} 0\\ 0,&{}\sigma ^2_{\mathcal {T}}I_{n_{\mathcal {T}}} \end{bmatrix}$$ where $\sigma ^2_{\mathcal {S}}$ and $\sigma ^2_{\mathcal {T}}$ are the source and target noise terms, respectively, and $${\tilde{K}}=\begin{bmatrix} {\tilde{K}}_{\mathcal{S}\mathcal{S}}, &{}{\tilde{K}}_{\mathcal{S}\mathcal{T}}\\ {\tilde{K}}_{\mathcal{T}\mathcal{S}}, &{}{\tilde{K}}_{\mathcal{T}\mathcal{T}} \end{bmatrix}$$ is the overall covariance matrix of the *inv*TGP. In ${\tilde{K}}$, ${\tilde{K}}_{\mathcal{S}\mathcal{S}}$ and ${\tilde{K}}_{\mathcal{T}\mathcal{T}}$ are the kernel matrices of the data in the source and target tasks, respectively. ${\tilde{K}}_{\mathcal{S}\mathcal{T}}$ ($={\tilde{K}}_{\mathcal{T}\mathcal{S}}^\intercal$) is the kernel matrix across the data in the source and target datasets.

#### Parameter learning

One way to learn the (hyper-)parameters of the *inv*TGP would be to consider the joint distribution of source and target tasks^49^. This may however cause the model to bias towards the source task when the volume of target data is less than that of the source. Thus, in this paper, a two-stage training process is employed instead. In the first stage, the parameters of the standard covariance function $k(\cdot ,\cdot )$ are learned based on the target data $D_{{\mathcal {T}},j}$ alone by maximizing,

$$\begin{aligned} - \frac{1}{2} \; X_{{\mathcal {T}},j}^\intercal \; ({\tilde{K}}_{\mathcal{T}\mathcal{T}}+\sigma ^2_{\mathcal {T}}I_{n_{\mathcal {T}}})^{-1} \; X_{{\mathcal {T}},j} -\frac{1}{2} \; \log (|{\tilde{K}}_{\mathcal{T}\mathcal{T}}+\sigma ^2_{\mathcal {T}}I_{n_{\mathcal {T}}}|) + const. \end{aligned}$$

In the second stage, the parameters found for $k(\cdot ,\cdot )$ are kept fixed while searching for $\lambda _j$ that optimizes the following log marginal likelihood considering both the source and the target data,

$$\begin{aligned} - \frac{1}{2} \; \bigl [ X_{{\mathcal {S}},j}^\intercal , \; X_{{\mathcal {T}},j}^\intercal \bigl ]({\tilde{K}}+\Lambda )^{-1}\begin{bmatrix}X_{{\mathcal {S}},j}\\ X_{{\mathcal {T}},j}\end{bmatrix} - \frac{1}{2} \; \log (|{\tilde{K}}+\Lambda |) + const. \end{aligned}$$

Note that the training complexity of the second stage scales cubically with the size of the data, i.e., as ${\mathcal {O}}\bigl ((n_{\mathcal {S}} + n_{\mathcal {T}})^3\bigl )$, due to the need for inversion and the determinant of ${\tilde{K}}+\Lambda$.

### Product-of-*inv*TGPs for multi-source transfer

The cubic complexity poses a major challenge while extending the TGP model to multi-source transfer learning since the total data size grows rapidly with the number of sources. A full TGP would additionally involve the modeling of correlations between *all* (source-target and source-source) task pairs, such that the number of parameters to be learnt would grow as the square of the number of sources. This makes parameter optimization difficult as well.

To overcome these challenges, in this paper we adapt the factorized product-of-GP experts for alleviating the cubic training cost^28,50^ and arriving at a novel product-of-*inv*TGPs. A significant advantage of factorization is that it allows for massively distributed computations in model training and posterior inference. The *inv*TGPs learnt for all source-target pairs form independent components that are efficiently trainable on distributed hardware. *As a useful aside, the assumed independence of target decision variables implies even greater scope for parallelization.* What’s more, when limiting to sequential computations, the time complexity of the product-of-experts (PoE) scales only linearly with respect to the number of sources.

Beyond computational gains, the PoE offers a principled fusion of individual *inv*TGP predictive distributions. This can be shown as follows. For the $j^{th}$ target decision variable, let $\mu _{k,j}({{\textbf {w}}}_q)$ and $\sigma _{k,j}^2({{\textbf {w}}}_q)$ be the predicted mean and variance at query point ${{\textbf {w}}}_q$ of the *inv*TGP trained (as per the procedure in Section “Inverse TGPs for single-source transfer”) with the $k^{th}$ source $D_{{\mathcal {S}}_k,j}$ and the target data $D_{{\mathcal {T}},j}$. The product of $\gamma$ such Gaussian predictions is then proportional to a Gaussian with mean and variance given by,

6a $$\begin{aligned} \mu _{PoE,j}({{\textbf {w}}}_q)&= \sigma _{PoE,j}^2 \sum _{k=1}^{\gamma } \sigma _{k,j}^{-2}({{\textbf {w}}}_q) \mu _{k,j}({{\textbf {w}}}_q), \end{aligned}$$

6b $$\begin{aligned} \sigma _{PoE,j}^2({{\textbf {w}}}_q)&= 1/ \bigl (\sum _{k=1}^{\gamma } \sigma _{k,j}^{-2}({{\textbf {w}}}_q) \bigl ) . \end{aligned}$$

As indicated by Eq. (6), the PoE composes the final prediction taking into account each *inv*TGP’s predictive uncertainty. Lower predicted variances (indicating more confident/certain predictions) are more strongly weighted, leading to an intuitively sound fused prediction. Imagine a situation where a source $k'$ results in an *inv*TGP whose predictive variance is large, such that $\sigma _{k',j}^{-2}<< \sigma _{k,j}^{-2}, \forall k \ne k'$. This could happen if $\lambda _{k',j}$ is much smaller in magnitude than the source-target correlations uncovered by the other *inv*TGPs. In such cases, Eq. (6a) implies that the $k'$ term will vanish from the PoE aggregation, providing a fused prediction that depends only on those *inv*TGPs that are confident at ${{\textbf {w}}}_q$.

By replicating the PS learning and prediction procedure (as shown for the $j^{th}$ variable) for all $d_{{\mathcal {T}}}$ target decision space dimensions, a complete solution $\varvec{\mu }_{PoE}({{\textbf {w}}}_q)$ corresponding to query point ${{\textbf {w}}}_q$ is constructed.

### A generalized product-of-*inv*TGPs

The product-of-*inv*TGPs offers both computational and predictive advantages in the multi-source transfer setting. However, as the number of source datasets (or *inv*TGPs) increases, Eq. (6b) implies that the predicted variance of the PoE would quickly drop to zero, suggesting overconfident predictions^51^. This is undesirable, as well-calibrated uncertainty-aware prediction is a key to rationalizable human-machine interaction^26^. *An overconfident prediction could mislead a DM into adopting a solution where the PoE is confident but wrong*. To alleviate this issue, a tunable parameter $\beta$ can be introduced into Eq. (6) to form the following generalized PoE (gPoE) prediction,

7a $$\begin{aligned} \mu _{gPoE,j}({{\textbf {w}}}_q)&= \sigma _{gPoE,j}^2 \sum _{k=1}^{\gamma } \beta _k \sigma _{k,j}^{-2}({{\textbf {w}}}_q) \mu _{k,j}({{\textbf {w}}}_q), \end{aligned}$$

7b $$\begin{aligned} \sigma _{gPoE,j}^2({{\textbf {w}}}_q)&= 1/ \bigl (\sum _{k=1}^{\gamma } \beta _k \sigma _{k,j}^{-2}({{\textbf {w}}}_q)\bigl ), \end{aligned}$$

where $\sum _{k=1}^{\gamma } \beta _k = 1$. In our implementation we set $\beta _k = 1/\gamma$. This makes the aggregated mean in Eq. (7a) identical to Eq. (6a)—hence preserving the intuitively sound fused prediction—while preventing the predicted variance in Eq. (7b) from degenerating to zero for large $\gamma$.

### A summary of salient features

Inverse transfer learning through common objective spaces is what enables PE to maximally benefit from mutual information between heterogeneous source-target pairs. Here, we further recap some of the salient features of our approach brought by the generalized product-of-*inv*TGPs, supporting PS learning in small data regimes.

- *Computationally efficient multi-source transfer.* The method gives rise to a factorized training scheme where *inv*TGPs for all source-target pairs form independent components that are efficiently trainable on distributed hardware. Hence, given a fully parallel computation setup, the training complexity is limited only by the largest data size among all paired source-target datasets. The cubic complexity in the number of sources is overcome.
- *Uncertainty-aware fusion of predicted means.* The gPoE aggregation weights individual *inv*TGPs inversely to their predictive uncertainty. This leads to a fused prediction that depends more strongly on *inv*TGPs with low predicted variance (higher confidence), while adaptively weighing out those with large predicted variance.
- *Calibrated predicted variance.* The gPoE does not lead to overconfident predictions under increasing number of sources (*inv*TGP models), facilitating rationalizable human-machine interactions with models that know what they don’t know.

## Empirical analysis

The generalized product-of-*inv*TGPs is implemented using the GPyTorch library^52^. Our method is first verified on the pedagogical DTLZ 1-3 benchmarks^53^, with slight modifications to synthetically create different source and target MOPs. Modified DTLZ 1-3 with 4 to 7 objective functions are used to analyse the performance of the method under: i) increasing levels of (target) data scarcity, ii) varying source-target similarity, and iii) multi-source transfer. A set of computationally expensive MOPs from the lightweight composites manufacturing domain are considered next. The use-case establishes the validity of our assumption (of common objective spaces) and the practical applicability of the method in augmenting PE under small data by means of inverse transfer learning.

### Evaluation metrics for pareto estimation

To evaluate the quality of post-hoc PE, we consider two different metrics, namely, the *Inverted Generational Distance (IGD) Ratio* and the *Root Mean Square Error (RMSE)*. The two metrics capture distinctive attributes of the candidate solutions generated from the perspective of a DM with postponed preferences.

The IGD Ratio adapted from Giagkiozis and Fleming^21^ gives a broad understanding of the overall PF approximation capacity of PE. It quantifies the improvement in the quality of PF approximation before and after PE as,

8 $$\begin{aligned} IGD \; Ratio = \frac{IGD_{b}}{IGD_{a}}, \end{aligned}$$

where $IGD_{b}$ and $IGD_{a}$ are the IGD values before and after, respectively. A ratio of 1 indicates that the PF approximation has not improved despite PE, while a value greater than 1 provides a scalar indicator of the relative convergence and diversity improvement. Values less than 1 do not occur as $IGD_{a}$ combines the predicted points with the training points. We remind that the IGD is a measure of the Euclidean distance between elements in the approximated PF and the true PF^45^;

9 $$\begin{aligned} IGD = \frac{1}{|Y^*|} \; \sum _{q=1}^{|Y^*|} \min \{||{{\textbf {y}}}_q^* - {{\textbf {y}}}_1||_2,\ldots, ||{{\textbf {y}}}_q^* - {{\textbf {y}}}_{n_q}||_2\}, \end{aligned}$$

where $Y^* = \{{{\textbf {y}}}_1^*, {{\textbf {y}}}_2^*, \dots , {{\textbf {y}}}_{n_q}^*\}$ is a set of $n_q$ well-distributed reference points along the true PF and ${{\textbf {y}}}_1,{{\textbf {y}}}_2,\dots ,{{\textbf {y}}}_{n_q}$ are the set of approximate points generated as ${{\textbf {y}}}_q = {{\textbf {f}}}(\varvec{\mu }_{gPoE}({{\textbf {w}}}_q))$. A lower IGD is clearly better.

In contrast to the IGD Ratio, the RMSE provides a more fine-grained evaluation of the accuracy of PE on a test set of $n_q$ query points (e.g., those supplied by a DM) not contained in the training data. For benchmark functions whose analytical expressions are known, the RMSE value is measured in the objective space as per (10a). The error thus quantifies how closely PE is able to satisfy specific DM preferences articulated in the objective space. On the other hand, calculating exact objective function values for predicted solutions in real-world MOPs can call for expensive evaluations. To avoid this, the RMSE can be measured in decision space instead, as per (10b). The latter is meaningful when we consider a smooth one-to-one mapping between the PS and the PF. The two instantiations of the RMSE are stated as,

10a $$\begin{aligned} RMSE_{{\textbf {f}}}&= \sqrt{\frac{\sum _{q=1}^{n_q} ||{{\textbf {y}}}_q^*-{{\textbf {y}}}_q||_2^2}{n_q}}, \end{aligned}$$

10b $$\begin{aligned} RMSE_{{\textbf {x}}}&= \sqrt{\frac{\sum _{q=1}^{n_q} ||{{\textbf {x}}}_q^*-{{\textbf {x}}}_q||_2^2}{n_q}}, \end{aligned}$$

where ${{\textbf {x}}}_q^*$ and ${{\textbf {x}}}_q$ are the true and predicted solutions, respectively, given the $q^{th}$ query/test point ${{\textbf {w}}}_q$. Note, the predicted mean of the product-of-*inv*TGPs is taken as its point estimate for accuracy evaluation, i.e., ${{\textbf {x}}}_q = \varvec{\mu }_{gPoE}({{\textbf {w}}}_q)$. In addition to the above, we also use the *coefficient of determination* ($R^2$ statistic) to compare the proportion of variation in the output of interest that a model explains; a higher $R^2$ score suggests better performance. A maximum test $R^2$ of 1 occurs for perfect predictions, while an $R^2 < 0$ indicates that the model’s performance is worse than a constant function that always predicts the mean of the test data. That latter could occur when models are trained with very limited data, as shall be seen without transfer learning in the multidisciplinary process design use-case.

### Results on modified DTLZ benchmarks

We begin by modifying the DTLZ 1-3 benchmarks (denoted as DTLZ 1a - 3a) to create different problem instances with heterogeneous decision spaces. These problems make up source and target MOPs with common objective spaces and PF topology, but with varying characteristics of the PS. DTLZ 1a-3a take the general form^54^ of,

11 $$\begin{aligned}&\min _{{{\textbf {x}}}_I, {{\textbf {x}}}_{II}} \;\;\; {{\textbf {f}}}\bigl ({{\textbf {x}}}, s, g({{\textbf {x}}}_{II})\bigl ) = \bigr [f_1\bigl ({{\textbf {x}}}_I, s, g({{\textbf {x}}}_{II})),\ldots, f_m({{\textbf {x}}}_I, s, g({{\textbf {x}}}_{II})\bigl )\bigr ],\\&\quad s.t. \;\;\; 0\le x \le 1, \; \forall x \in \{{{\textbf {x}}}_I, {{\textbf {x}}}_{II}\}, \end{aligned}$$

where *m* is the number of objectives to be minimized. *d* is the total number of decision variables constituting ${{\textbf {x}}}_I= [x_1,\ldots, x_{m-1}]$ and ${{\textbf {x}}}_{II}=[x_m,\ldots,x_d]$ with $d\ge m$, and *s* changes the distribution of the non-dominated solutions.

The objective values of DTLZ 1a are given by Eq. (12a) while those of DTLZ 2a and 3a are given by Eq. (12b);

12a $$\begin{bmatrix} f_1\\ f_2\\ \ldots \\ f_{m-1}\\ f_{m}\\ \end{bmatrix}^\intercal = 0.5 \bigl (1+g({{\textbf {x}}}_{II})\bigl ) \begin{bmatrix} {x_1}^s \; {x_2}^s \ldots \; {x_{{m-1}}}^s \\ {x_1}^s \; {x_2}^s \ldots \; (1-{x_{m-1}}^s)\\ \ldots \\ {x_1}^s \; (1-{x_2}^s)\\ (1-{x_1}^s)\\ \end{bmatrix}^\intercal ,$$

12b $$\begin{bmatrix} f_1\\ f_2\\ \ldots \\ f_{m-1}\\ f_{m}\\ \end{bmatrix}^\intercal = \bigl (1+g({{\textbf {x}}}_{II})\bigl ) \begin{bmatrix} cos(\frac{{x_1}^s\,\pi }{2})\ldots \; cos(\frac{{x_{m-2}}^s\,\pi }{2}) \; cos(\frac{{x_{m-1}}^s\,\pi }{2})\\ cos(\frac{{x_1}^s\,\pi }{2})\ldots \; cos(\frac{{x_{m-2}}^s\,\pi }{2}) \; sin(\frac{{x_{m-1}}^s\,\pi }{2})\\ cos(\frac{{x_1}^s\,\pi }{2})\ldots \; sin(\frac{{x_{m-1}}^s\,\pi }{2})\\ \ldots \\ sin(\frac{{x_{m-1}}^s\,\pi }{2})\\ \end{bmatrix}^\intercal ,$$

where *s* is set to 1 for all target MOPs, and $s \in (0, 1)$ for source MOPs to simulate different degrees of source-target similarity. A value of *s* closer to 1 indicates higher similarity.

The function $g({{\textbf {x}}}_{II})$ in Eq. (12) is given by Eq. (13a) for DTLZ 2a and Eq. (13b) for DTLZ 1a and 3a;

13a $$\begin{aligned} g({{\textbf {x}}}_{II})&= \sum _{x_j \in {{\textbf {x}}}_{II}}(x_j - p_j)^2, \end{aligned}$$

13b $$\begin{aligned} g({{\textbf {x}}}_{II})&= 100 \; |{{\textbf {x}}}_{II}| \sum _{x_j \in {{\textbf {x}}}_{II}} \bigr [(x_j-p_j)^2 -cos\bigl (2\pi (x_j-p_j)\bigl )\bigr ], \end{aligned}$$

where $p_j=0.5$ for all target MOPs, and $p_j = \frac{j-|{{\textbf {x}}}_I|}{k |{{\textbf {x}}}_{II}|}$ for all source MOPs $k=1,2,\ldots,\gamma$.

To produce the source and target datasets for DTLZ 1a-3a, the NSGA-III algorithm from the pymoo library^55^ is run to generate the PF and PS approximations. All results of post-hoc PE are averaged over 20 runs of GP training with the squared exponential covariance function optimized by Adam^56^. We consider heterogeneous source and target MOPs with $d_{{\mathcal {S}}} = 10$ and $d_{{\mathcal {T}}} = 12$ decision variables. Table 1 shows the experimental settings where the amount of source data (per source MOP) is about twice that of available target data. The set of $n_q$ query/test points of potential interest to a DM are evenly spaced along the projected hyperplane in the objective space. $n_q$ is relatively large, allowing for rigorous evaluation of Pareto approximation capacity as indicated by the IGD Ratio.

**Table 1:** Experiment settings used for the size of the source data ($n_{\mathcal {S}}$), the target data ($n_{\mathcal {T}}$), and the number of query points ($n_q$) employed for testing post-hoc PE on the DTLZ 1a-3a benchmarks with 4 to 7 objective functions.

DTLZ 1a-3a	7-Obj	6-Obj	5-Obj	4-Obj
$n_{\mathcal {S}}$	413	498	246	236
$n_{\mathcal {T}}$	161	246	135	108
$n_q$	5005	2002	1820	1330

#### Impact of target data scarcity on pareto set learning

The effect of small target data in high-dimensional optimization domains is illustrated on DTLZ 1a-3a with 4 and 7 objectives. The numbers for $n_{\mathcal {T}}$ in Table 1 indicate 100% of the target data available for training the inverse machine learning model. The amount of target data utilized is gradually reduced to 50% and 25% to study the consequences on the quality of PE.

**Figure 3:**
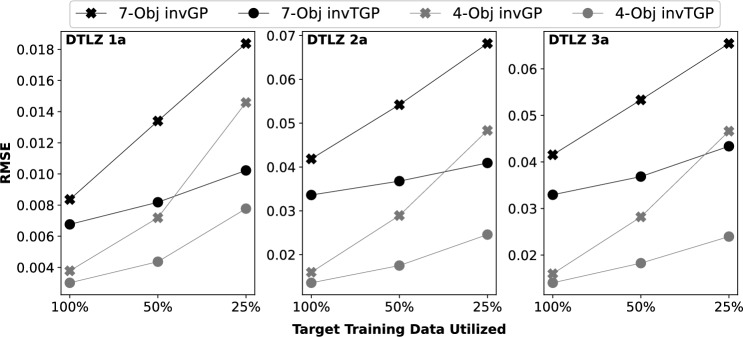
Accuracy of PE measured in $RMSE_{{\textbf {f}}}$ (y-axis) for different amounts of target training data utilized (x-axis). Results for DTLZ 1a to 3a with 7 objectives (black line) or 4 objectives (grey line) are presented. The marker “o” and “x” represent PS learning by *inv*TGP with single-source transfer and *inv*GP without transfer, respectively.

From Fig. 3, a monotonic worsening (increasing) trend is observed in the RMSE value as the amount of target data is decreased. This is not surprising. Interestingly, Fig. 3 shows that by transfer learning from a correlated source MOP with $s=0.9$, the *inv*TGP is able to resist the negative effects of data scarcity to a large extent. In particular, the RMSE is lowered by up to $\sim$50% when compared to the *inv*GP with no transfer.

The $R^2$ scores were also computed from the obtained results. Both *inv*GP and *inv*TGP achieved consistently high scores across the benchmark MOPs. The worst case $R^2$ performance of *inv*GP was $\sim$0.94 while that of *inv*TGP was even higher at $\sim$0.98, demonstrating the usefulness of PS learning in general.

#### Effect of source-target similarity

**Figure 4:**
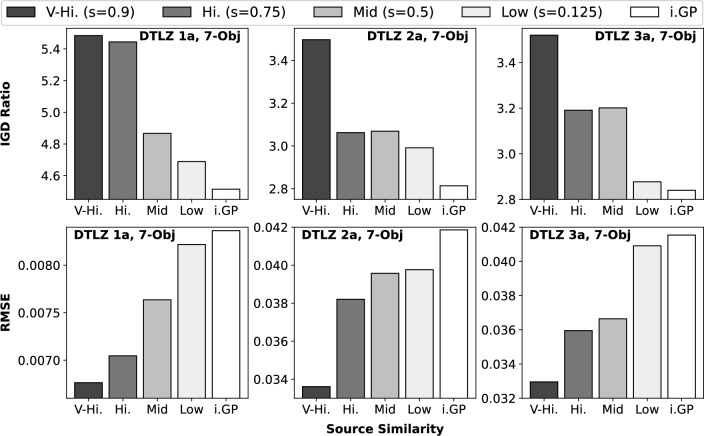
Quality of PE measured in IGD Ratio (y-axis, top row) and $RMSE_{{\textbf {f}}}$ value (y-axis, bottom row) with different levels (*s*) of source-target similarity (x-axis). i.GP refers to the baseline *inv*GP with no transfer; all other results are from the *inv*TGP.

The second set of experiments for DTLZ 1a-3a aims at investigating the performance of *inv*TGP under different levels of source-target similarity, compared against the baseline case of *inv*GP with no transfer. The quality of PE measured by the IGD Ratio and the RMSE value are depicted in Figure 4. From the results, not only does the *inv*TGP outperform the *inv*GP, but also as the source-target similarity increases, the quality of PE tends to improve consistently for the *inv*TGP. This improvement makes intuitive sense and indicates that the *inv*TGP successfully leverages the correlation between the target task and the different source MOPs, transferring the external information weighted by $\lambda _j$ in (4) to augment its performance.

#### Utilizing multi-source transfers

**Figure 5:**
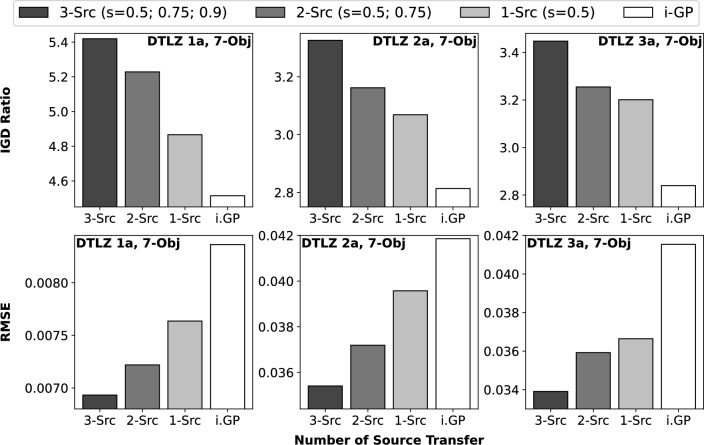
Quality of PE measured in IGD Ratio (y-axis, top row) and $RMSE_{{\textbf {f}}}$ value (y-axis, bottom row) with decreasing number of source MOPs (x-axis) for DTLZ 1a to 3a with 7 objectives. i.GP refers to the baseline *inv*GP with no transfer.

The final set of experiments with benchmark functions investigates the performance of the generalized product-of-*inv*TGPs under multi-source transfer. Given a high 7-D objective space, Fig. 5 shows that the performance of the model improves substantially when additional data from source MOPs with larger source-target correlation are introduced. Note that in most practical situations, inter-task correlations would not be known beforehand. Hence, an important property of an effective transfer learning algorithm is to be able to selectively exploit useful information sources without the need for a human in the loop, while curbing harmful negative transfer from unrelated data. The aggregation equations Eqs. (6a) and (7a) suggest this to be the case in theory. The experimental results substantiate that the model is indeed able to fuse information from all available sources to construct more accurate predicted solutions.

The experiments above are extended to DTLZ 1a-3a with 4 to 7 objective functions. Tables 2 and 3 present the detailed results, showcasing that the product-of-*inv*TGPs often leads to superior PE. Interestingly, monotonically improving performance is observed here as the number of source MOPs increases. Table 2 includes yet another commonly used inverse machine learning model, namely, the inverse radial basis function neural network (*inv*RBFNN), as a baseline for comprehensive comparison. The network structure and hyperparameters of the *inv*RBFNN were implemented by us according to the specifications by Giagkiozis and Fleming^21^. The *inv*RBFNN was found to under-perform relative to the *inv*GP and hence has been left out from the engineering case-study presented next.

### A multidisciplinary process design use-case

Here, we apply the generalized product-of-*inv*TGPs model to a practical use-case in the manufacturing of lightweight fiber-reinforced polymer (FRP) composites. Two distinct manufacturing techniques are considered, naturally forming source and target tasks in a transfer learning setting; detailed descriptions of these techniques can be found in the work by Gupta^57^. The first, labelled resin transfer moulding (RTM), involves placing a fibrous reinforcement inside a mould cavity whose geometry is precisely machined according to the FRP part to be produced. The mould is completely closed at the start of the manufacturing cycle, fully compressing the dry fibres to the desired fibre volume fraction. The mould is then heated to an operation temperature at which liquid thermosetting resin is injected into it at high pressure until the cavity is filled. After mould filling, the part rests and cures under controlled temperature until the liquid resin sufficiently solidifies. The two phases (filling and curing) of the manufacturing cycle form a multidisciplinary design problem, deeply coupled by the thermal conditions induced in the part at the end of filling. A candidate process design is therefore evaluated by first running the mould filling simulation code, the output of which gives the initial thermal condition for the curing simulation.

**Table 2:** Quality of PE measured in IGD Ratio given 1 source (*s* = 0.5), 2 sources (*s* = 0.5, 0.75) or 3 sources (*s* = 0.5, 0.75, 0.9) for transfer. Values in bold mark the best averaged performance for a given target MOP over 20 independent PE runs. Values in brackets represent standard deviations in performance over these runs.

Pareto Estimator	# Objective functions			
	7-Obj	6-Obj	5-Obj	4-Obj
invTGPs	IGD Ratio for DTLZ 1a			
3 Sources	**5**.**42** (0.01)	**7**.**51** (0.01)	**7**.**98** (0.02)	**12**.**13** (0.16)
2 Sources	5.23 (0.01)	7.29 (0.01)	7.50 (0.02)	10.62 (0.10)
1 Source	4.87 (2e−3)	6.90 (0.01)	6.96 (0.01)	9.29 (0.10)
invGP	4.51 (5e−8)	6.95 (1e−7)	6.83 (2e−7)	8.55 (4e−7)
invRBFNN	3.57 (2e−6)	1.42 (4e−5)	1.30 (9e−6)	1.10 (1e−4)
invTGPs	IGD Ratio for DTLZ 2a			
3 Sources	**3**.**33** (0.02)	**4**.**33** (0.03)	**5**.**72** (0.04)	**6**.**46** (0.02)
2 Sources	3.16 (0.02)	4.04 (0.03)	5.36 (0.02)	5.66 (0.01)
1 Source	3.07 (0.02)	4.00 (0.02)	5.22 (0.01)	5.56 (0.12)
invGP	2.81 (1e−07)	3.63 (2e−07)	5.19 (3e−07)	5.48 (4e−07)
invRBFNN	1.66 (2e−10)	1.70 (6e−8)	2.68 (9e−8)	2.29 (7e−4)
invTGPs	IGD Ratio for DTLZ 3a			
3 Sources	**3**.**45** (0.02)	**4**.**07** (0.04)	**5**.**16** (0.01)	**6**.**22** (0.03)
2 Sources	3.25 (0.02)	3.83 (0.07)	5.13 (0.01)	5.99 (0.04)
1 Source	3.20 (0.02)	3.43 (0.01)	4.70 (4e−3)	5.85 (0.06)
inv**GP**	2.84 (1e−7)	3.27 (0.01)	4.26 (3e−7)	5.55 (4e−7)
invRBFNN	1.72 (6e−10)	1.41 (5e−7)	1.63 (1e−6)	1.21 (6e−4)

**Table 3:** Quality of PE measured in $RMSE_{{\textbf {f}}}$ value given 1 source (*s* = 0.5), 2 sources (*s* = 0.5, 0.75) or 3 sources (*s* = 0.5, 0.75, 0.9) for transfer. Values in bold mark the best averaged performance for a given target MOP over 20 independent PE runs. Values in brackets represent standard deviations in performance over these runs.

Pareto Estimator	# Objective functions			
	7-Obj	6-Obj	5-Obj	4-Obj
invTGPs	RMSE $\cdot 1\text {E}-2$ for DTLZ 1a			
3 Sources	**0**.**69** (1E−3)	**0**.**46** (3E-4)	**0**.**45** (9E-4)	**0**.**24** (3E-3)
2 Sources	0.72 (7E-4)	0.48 (6E-4)	0.47 (1E-3)	0.27 (3E-3)
1 Source	0.76 (3E-4)	0.49 (4E-4)	0.52 (9E-4)	0.33 (0.01)
invGP	0.84 ( 1E-8)	0.48 (6E-9)	0.56 (8E-8)	0.38 (3E-8)
invTGPs	RMSE $\cdot 1\text {E}-2$ for DTLZ 2a			
3 Sources	**3**.**54** (0.02)	**2**.**29** (0.01)	**1**.**85** (0.01)	**1**.**33** (2E-3)
2 Sources	3.72 (0.02)	2.43 (0.02)	1.95 (4E-3)	1.48 (2E-3)
1 Source	3.96 (0.05)	2.46 (0.01)	2.02 (4E-3)	1.54 (0.02)
invGP	4.19 (1E-7)	2.77 (1E-7)	2.10 (9v8)	1.60 (1E-7)
invTGPs	RMSE $\cdot 1\text {E}-2$ for DTLZ 3a			
3 Sources	**3**.**39** (0.01)	**2**.**46** (0.03)	**2**.**05** (4E−3)	**1**.**41** (0.01)
2 Sources	3.59 (0.02)	2.60 (0.04)	2.07 (4E−3)	1.46 (0.01)
1 Source	3.66 (0.02)	2.85 (0.05)	2.22 (1E−3)	1.46 (0.01)
invGP	4.15 (1E−7)	3.05 (5E−8)	2.51 (1E−7)	1.60 (1E−7)

Compression resin transfer moulding (CRTM) is an alternate technique that can shorten manufacturing cycle time but usually at the cost of larger peripheral equipment. This is achieved by a slight modification to the filling phase of the RTM cycle. Specifically, in CRTM, the mould is only partially closed before resin injection, reducing the resistance to the resin’s flow. Full closure to the final fibre volume fraction occurs after fibre wetting with the required volume of liquid resin. The need for larger equipment (e.g., hydraulic press) thus originates from having to jointly compress the resin + fibre system.

**Figure 6:**
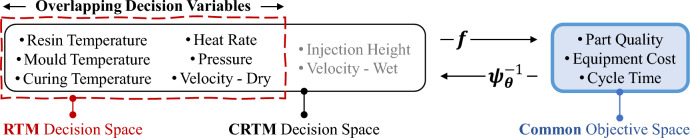
The common 3-D objective space and the overlapping decision variables of the heterogeneous RTM and CRTM manufacturing processes.

Despite the difference in the design (and hence the decision space) of the RTM and CRTM processes, their objective functions from a manufacturing standpoint are identical. In both cases the goal is to maximize *part quality* while minimizing *equipment cost* and *cycle time*, forming MOPs with 3-D objective spaces as descried by Gupta et al.^20^. The finite element simulation codes for approximating these objectives are generally expensive, allowing small but high-quality data to be generated. The scenario thus perfectly encompasses the assumptions made in this paper. Figure 6 illustrates the common objective space and the heterogeneous but overlapping decision spaces of the MOPs under consideration. The six overlapping decision variables pertain to the thermal conditions of the resin and the mould (namely, *Resin Temperature, Mould Temperature, Heat Rate, Curing Temperature*), liquid injection pressure (*Pressure*) and the dry fibre compression velocity (*Velocity - Dry*). CRTM introduces two additional decision variables, namely, the *Injection Height* of the mould prior to resin injection and the wet fibre compression velocity (*Velocity - Wet*).

We consider MOPs arising from the manufacture of FRP parts of circular geometry made of glass-fibre reinforced epoxy. The plates are of 1 m diameter with a central injection hole of 20 mm. The final part fibre volume fraction is either 35% or 40%. By accounting for two different manufacturing processes we get a total of four MOPs: R35, R40, C35, and C40. Here R represents RTM, C represents CRTM, and the numerical value represents the part’s final fibre volume fraction. At the end of multi-objective optimization runs for each task, datasets containing 500 optimized solution samples are collected. For assessing post-hoc PE, the target dataset is further divided into training and testing splits of 10 and 490 points, respectively, serving as an example of machine learning under expensive and extremely small data. The amount of source data (per source MOP) is taken to be 50 points. Given the computational expense of running evaluations at a large number of query points, only the $RMSE_{{\textbf {x}}}$ value on the test set is used as the metric for comparison herein.

Table 4 shows the accuracy of PE under different source-target combinations. The high degree of overlap in the objective and decision spaces of related manufacturing tasks intuitively suggests the existence of transferrable information between them. It is therefore not surprising that both single-source and multi-source transfer learning with *inv*TGPs show benefits over the standard *inv*GP model trained only on limited target data. In the case of R35 as target task, a reduction in RMSE of up to $\sim$17% is achieved as a consequence of transfer. For R40, we see that no transfer leads to a negative $R^2$ score given the extremely small target training data, whereas $R^2$ is always positive across all cases of post-hoc PE with *inv*TGPs. Unlike in the case of benchmark functions, the best averaged performance in Table 4 is not achieved when all source data is utilized for multi-source transfers. This observation warrants future investigation. *It is however striking that multi-source transfer always leads to significantly better predictions than the least performant single-source* *invTGPs*, thus motivating joint utilization of all available sources in practical scenarios where source-target correlations may be a priori unknown.

**Table 4:** Quality of PE measured in $RMSE_{{\textbf {x}}}$ and $R^2$ values for the composite part manufacturing use-case. Values in bold mark the best averaged performance for a given target MOP over 20 independent PE runs. Transfer learning consistently outperforms no-transfer. Strikingly, multi-source transfer utilizing *all* sources (last row of the table) always leads to significantly better performance (lower RMSE and higher $R^2$) than the least performant single-source *inv*TGPs.

Source task(s)	Target task							
	C35		C40		R35		R40	
	RMSE $\cdot 1\text {E}-2$	$R^2$ $\%$	RMSE $\cdot 1\text {E}-2$	$R^2$ $\%$	RMSE $\cdot 1\text {E}-2$	$R^2$ $\%$	RMSE $\cdot 1\text {E}-2$	$R^2$ $\%$
No Transfer	19.36 (0)	23.78 (0)	22.56 (1E−14)	4.48 (1E−15)	25.97 (6E−15)	24.53 (6E−15)	28.68 (6E−15)	−5.9 (7E−16)
C35	–	–	21.49 (6E−2)	11.44 (1.25)	25.13 (6E−2)	30.17 (5E−1)	27.00 (1E−1)	1.70 (7E−1)
C40	18.43 (6E−2)	28.67 (4E−1)	–	–	26.18 (3E−1)	25.84 (1.06)	**25**.**79** (4E−1)	**7**.**86** (2.20)
R35	18.21 (3E−2)	19.36 (5E−1)	22.02 (5E−2)	8.78 (1.24)	–	–	27.25 (3E−1)	0.21 (3E−1)
R40	**18**.**02** (5E−2)	**29**.**95** (4E−1)	21.87 (2E−1)	9.59 (1.44)	**21**.**74** (6E−1)	**41**.**87** (1.88)	–	–
C35-C40	–	–	–	–	25.53 (9E−2)	28.71 (4E−1)	25.92 (1E−1)	6.98 (1.00)
C35-R35	–	–	21.61 (7E−2)	11.11 (8E−1)	–	–	26.83 (2E−1)	2.26 (1.00)
C35-R40	–	–	**21**.**45** (8E−1)	**12**.**14** (1.00)	23.16 (4E−1)	37.20 (1.30)	–	–
C40-R35	18.27 (3E−2)	29.35 (3E−2)	–	–	–	–	26.31 (2E−1)	5.06 (1.31)
C40-R40	18.13 (6E−2)	29.76 (3E−2)	–	–	23.58 (4E−1)	35.81 (1.36)	–	–
R35-R40	18.27 (3E−2)	29.86 (3E−1)	21.91 (1E−1)	9.42 (1.08)	–	–	–	–
All	18.14 (4E−2)	29.76 (2E−1)	21.61 (6E−2)	11.23 (8E−1)	24.00 (3E−1)	34.34 (1E−1)	26.25 (1E−1)	5.27 (9E−1)

## Conclusion

This paper takes an important step towards effective human-machine interactions in multi-objective decision-making, particularly in high-dimensional/expensive optimization domains characterized by data scarcity. To this end, a novel methodology for PS learning under small data to recover non-dominated solutions along sparsely populated PFs is proposed. Our method is the first to explore the concept of multi-source, inverse transfer Gaussian processes (*inv*TGPs) for post-hoc *Pareto estimation* (PE), leveraging MOPs with common objective spaces to maximally utilize information between heterogeneous source-target pairs. To avoid computational bottlenecks arising from a large number of source datasets, a factorized product-of-experts procedure is put forth. The advantage of the adapted product-of-experts is that it not only facilitates massively distributed training, but also gives rationalizable predictive distributions that fuse together *inv*TGPs drawn from multiple sources to augment PE in the *target* optimization task at hand.

The resulting product-of-*inv*TGPs model is put through extensive empirical tests. Experiments are carried out on modified DTLZ benchmarks as well as on practical MOPs with computationally expensive, multidisciplinary evaluation data. The results obtained are promising and clearly highlight the benefits of jointly utilizing all available source datasets for transfer, especially in complex real-world scenarios where source-target correlations may not be known beforehand.

A major focus of this work has been on PE in high-dimensional objective spaces that lead to sparse PF approximations. Future work shall consider the curse of dimensionality even in decision space, with dimensionality reduction techniques (to discover low-dimensional, piecewise continuous manifolds on which Pareto optimal solutions tend to lie^35^) for effective learning of the inverse model(s). We also foresee transfer learning-enabled PS learning to be coupled with MOP solvers in the *online PE* mode, potentially illuminating new kinds of multi-objective transfer optimization algorithms.

## Data Availability

Correspondence and requests for materials should be addressed to A.G.
